# Intake to Production Ratio: A Measure of Exposure Intimacy for Manufactured Chemicals

**DOI:** 10.1289/ehp.1204992

**Published:** 2012-09-25

**Authors:** William Nazaroff, Charles J. Weschler, John C. Little, Elaine A. Cohen Hubal

**Affiliations:** 1 Department of Civil and Environmental Engineering, University of California, Berkeley, Berkeley, California, USA; 2 Environmental and Occupational Health Sciences Institute, University of Medicine and Dentistry of New Jersey and Rutgers University, Piscataway, New Jersey, USA; 3 International Centre for Indoor Environment and Energy, Technical University of Denmark, Lyngby, Denmark; 4 Charles E. Via Jr. Department of Civil and Environmental Engineering, Virginia Tech, Blacksburg, Virginia, USA; 5 National Center for Computational Toxicology, U.S. Environmental Protection Agency, Research Triangle Park, North Carolina, USA

**Keywords:** bioavailability, bisphenol A, *p*-dichlorobenzene, pentachlorophenol, phthalate, screening, triclosan

## Abstract

Background: Limited data are available to assess human exposure to thousands of chemicals currently in commerce. Information that relates human intake of a chemical to its production and use can help inform understanding of mechanisms and pathways that control exposure and support efforts to protect public health.

Objectives: We introduce the intake-to-production ratio (IPR) as an economy-wide quantitative indicator of the extent to which chemical production results in human exposure.

Methods: The IPR was evaluated as the ratio of two terms: aggregate rate of chemical uptake in a human population (inferred from urinary excretion data) divided by the rate that chemical is produced in or imported into that population’s economy. We used biomonitoring data from the U.S. Centers for Disease Control and Prevention along with chemical manufacturing data reported by the U.S. Environmental Protection Agency, as well as other published data, to estimate the IPR for nine chemicals in the United States. Results are reported in units of parts per million, where 1 ppm indicates 1 g of chemical uptake for every million grams of economy-wide use.

Results: Estimated IPR values for the studied compounds span many orders of magnitude from a low of 0.6 ppm for bisphenol A to a high of > 180,000 ppm for methyl paraben. Intermediate results were obtained for five phthalates and two chlorinated aromatic compounds: 120 ppm for butyl benzyl phthalate, 670 ppm for di(2-ethylhexyl) phthalate, 760 ppm for di(*n*-butyl) phthalate, 1,040 ppm for *para*-dichlorobenzene, 6,800 ppm for di(isobutyl) phthalate, 7,700 ppm for diethyl phthalate, and 8,000–24,000 ppm (range) for triclosan.

Conclusion: The IPR is well suited as an aggregate metric of exposure intensity for characterizing population-level exposure to synthesized chemicals, particularly those that move fairly rapidly from manufacture to human intake and have relatively stable production and intake rates.

Exposures to manufactured chemicals can pose health risks (Collins et al. 2008; Rudén and Hansson 2010). Effective management of these risks requires knowledge not only about chemical toxicities but also about human exposures. Tracing exposure pathways from sources to receptors is challenging. Innovative approaches are needed to leverage exposure information that is available for only a small subset of the thousands of chemicals in commerce (Egeghy et al. 2012). Multimedia fate and transport models have been combined with metrics such as the intake fraction (iF) for large-scale assessments (Bennett et al. 2002a). Proximity between chemical releases and exposed populations is emerging as a parameter of key influence for assessing human intake (Manneh et al. 2010). Methods for characterizing the degree of exposure intimacy of human populations to manufactured chemicals could improve understanding of exposure pathways and assist in studies that aim to understand biologically relevant exposures (i.e., those associated with a disease) (Cohen Hubal 2009).

The existence of two national data sets in the United States creates an opportunity for computing a new aggregate exposure metric for certain chemicals, including several species that are of current environmental health interest. The Chemical Data Reporting system (formerly known as Inventory Update Reporting), maintained by the U.S. Environmental Protection Agency (EPA), collects and manages data concerning the manufacture and importation of industrial chemicals (U.S. EPA 2012). The National Report on Human Exposure to Environmental Chemicals, a project of the U.S. Centers for Disease Control and Prevention (CDC), employs biomonitoring to characterize exposure of the U.S. population to environmental chemicals (CDC 2012). We used data from these two systems to compute a dimensionless measure of exposure intensity, the “intake-to-production ratio” (IPR). In the present study, we estimated IPR values for the U.S. population for nine organic chemicals: bisphenol A (BPA), five phthalates [butyl benzyl phthalate (BBzP), di(2-ethylhexyl) phthalate (DEHP), diethyl phthalate (DEP), di(isobutyl) phthalate (DiBP), and di(*n*-butyl) phthalate (DnBP)], para-dichlorobenzene (DCB), methyl paraben (MP), and triclosan. The resulting IPR values span a remarkably broad range—more than five orders of magnitude for these nine chemicals. We explore the significance of the results, the limitations of the concept, and how the IPR might contribute to an improved understanding of population-level exposure to manufactured chemicals.

## Methods

Aggregate exposure metrics should provide useful information about exposure, should be applicable to many chemicals, and should be economical to implement. Population-level exposure metrics are required to extend molecular and cellular-level insights to inform risk assessment and decision making (Ankley et al. 2010). One such metric is introduced here: the IPR. We define the IPR to be the aggregate rate of chemical uptake in a human population (estimated here based on urinary excretion data) divided by the rate at which that chemical is produced in or imported into that population’s economy. The IPR is a dimensionless ratio that we report here using a parts-per-million (10^–6^) modifier. For example, an IPR of 1.0 ppm indicates that, on average, for every million grams of the chemical entering the economy, 1 g enters the aggregate human population served by that economy. The IPR does not reflect the distribution of exposures within a population: 1 g of uptake by one individual person yields the same IPR as 1 mg of uptake by each of 1,000 individuals.

The IPR is intended as a magnitude estimate that characterizes the degree of exposure intimacy between a chemical and a population. Knowledge about exposure pathways is not required for estimating IPR values. However, knowledge of the IPR can help inform investigations that aim to elucidate important exposure pathways. The IPR is simple in concept, but data availability is a limiting constraint for its use today. In principle, the IPR might be developed and applied for many manufactured chemicals and for a variety of economy-based populations. In the present study, we emphasized chemicals that are commonly used in consumer products, in building materials or furnishings, or in other products that might lead to widely distributed, close contact with populations. One broad class of interest is the semivolatile organic compounds (SVOCs) (Weschler and Nazaroff 2008), many species of which are commonly found indoors (Rudel et al. 2003). Some SVOCs have associated health concerns owing to the known or suspected endocrine-disrupting and/or carcinogenic properties (Rudel and Perovich 2009; Rudel et al. 2003). The specific demonstrations in this study for contemporary exposures were limited to a set of chemicals that do not bioaccumulate and for which urinary excretion, either of the primary compound (whether conjugated or not) or of one or more metabolites, is the dominant biological loss pathway. However, these conditions are not inherent limitations for the IPR metric. We illustrate this point in the discussion by estimating an IPR for pentachlorophenol from an historic interpretation incorporating multimedia fate and transport modeling as a basis for estimating population intake.

*Production data.* The specific demonstration in this study focused on the general U.S. population. Production data were mainly obtained from the U.S. EPA’s Inventory Update Reporting and Chemical Data Reporting system (U.S. EPA 2012), whose purpose is

to collect quality screening-level, exposure-related information on chemical substances and to make that information available for use by the U.S. EPA and, to the extent possible, to the public.

Until recently, the system was known as Inventory Update Reporting (IUR). IUR data have been historically reported at 4-year intervals, starting in 1986. The most recent IUR data, for 2006, summarized production and imports for calendar year 2005. Under the reporting rules (U.S. EPA 2008),

manufacturers and importers producing 25,000 pounds or more of a reportable chemical substance must report the identity of the chemical substance and basic manufacturing information.

In the 2006 report, the IUR compiled data reported by 1,541 companies covering 3,827 sites and 6,200 chemicals (U.S. EPA 2008). Total production and importation rates may be higher than used in the present analysis because of the exclusion of operations smaller than the reporting limit. That cause of bias appears to be only potentially significant for MP (reported production of < 0.5 million lb/year) and DiBP (0.5–1 million lb/year). For all other chemicals, the cumulative production rates exceed the reporting limit by at least two orders of magnitude.

For phthalates, we obtained production data directly from the U.S. EPA IUR. For BPA, only a lower bound was reported by IUR (specifying that > 1 billion lb/year was produced in each report from 1990 to 2006). A summary risk appraisal for BPA provided a more precise value [National Toxicology Program (NTP) 2007]. The most recent IUR does not contain data for triclosan; instead, we used the most recently reported national data, which are from the 1998 IUR (Fang et al. 2010). For the period 1986–1998, triclosan production increased substantially. Allowing that the growth might have continued, we treat the 1998 production rate as a lower bound and estimate an upper bound on production in 2002 by fitting an exponential growth rate to the data for 1986–1998.

In cases where production is reported to occur only within a specified range, we used the geometric mean (GM) of the upper and lower bounds of the range as the central best estimate of total production. Because many of the ranges are broad, uncertainty in production dominates total uncertainty of IPR estimates for most chemicals. For chemicals with multiplicatively large ranges, the production uncertainty is estimated as the multiplicative factor that links the central best estimate to the upper end of the reported range. For details, see Supplemental Material, pp. 1–2 (http://dx.doi.org/10.1289/ehp.1204992).

To estimate the per capita daily production, the central estimate aggregate annual production was converted to grams per person per day by means of unit conversion and by dividing by the estimated total U.S. population, using U.S. census data for the appropriate year. Detailed results showing per capita production rate estimates for the period 1986–2006 are presented in Supplemental Material, Tables S1 and S2 (http://dx.doi.org/10.1289/ehp.1204992).

*Intake estimates based on urinary excretion data.* Biomonitoring-based exposure information is being gathered by the CDC for the U.S. population (CDC 2012). The current analyte list comprises 212 chemicals, and we used data from the February 2012 update. For the IPR estimates reported here, we have matched—to the extent possible—the timing of urinary excretion sampling with the timing of production data. We used the CDC data for the U.S. population ≥ 6 years of age to estimate arithmetic mean (AM) levels of target analytes in urine, expressed in micrograms of analyte per gram of creatinine.

To elaborate, our goal in using the CDC biomonitoring data was to estimate the cumulative excretion rate of the chemical of interest or its key metabolite(s) summed over the entire U.S. population. For each sampling period, the CDC reports GMs as well as the 50th, 75th, 90th, and 95th percentile results for the U.S. population ≥ 6 years of age. For the compounds of interest here, the distributions of analyte concentrations in urine exhibit substantial skewing. So, although the GM represents well the central tendency of individual excretion rates, it is substantially below the population mean value. To estimate a mean from the tabulated data, we fit log-normal distributions to the four reported percentile values to obtain best-fit GMs and geometric SDs (GSDs). The fitting was done as a least-squares linear regression of the logarithm of the measured value against the *z*-score that corresponds to the indicated percentile value. An *r*^2^ value (coefficient of determination) for the regression was computed as an indicator of goodness of fit of the log-normal form to the 50th–95th percentile results. Given the fitted GM and GSD, the AM was computed from Equation 1, which holds for log-normal distributions:


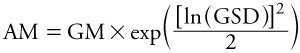
[1]

Creatinine-corrected excretion data for the whole sampled population were used for the time period that corresponded most closely to the year for which production data were available. Table 1 summarizes the resulting estimates of AM excretion rates of target analytes. The ratio of AM to GM for most of the chemicals varies over a range from 1.5 (MiBP) to 3.2 [monoethyl phthalate (MEP)]. However, for 2,5-dichlorophenol (DCP) and triclosan, the ratios are much higher, 11.8 and 13.5, respectively, reflecting the very large ranges of levels in urine, with the 95th percentile concentration exceeding the medians by factors of approximately 40 in each case.

**Table 1:** Computed AM analyte levels in urine (µg/g creatinine) for the U.S. population (≥ 6 years of age).*^a^*

Percentile						
Analyte	Year	50th	75th	90th	95th	GM	GSD	r2	AM
BPAb	2003–2004	2.50	4.29	7.67	11.2	2.43	2.5	1.00	3.7
DCP	2005–2006	7.32	20.4	89.3	292.0	6.0	9.2	0.98	71
MBzP	2005–2006	8.24	15.3	30.2	47.4	7.92	2.9	1.00	14
MEHP	2005–2006	2.61	5.69	13.7	30.1	2.38	4.3	0.98	6.9
MEHHP	2005–2006	21.4	46.1	117.0	235.0	19.6	4.2	0.99	56
MEOHP	2005–2006	13.5	28.9	77.7	144.0	12.4	4.2	0.99	35
MECPP	2005–2006	32.2	67.5	168.0	290.0	30.2	3.8	0.99	75
MEP	2005–2006	92.3	242.0	625.0	1140.0	90.0	4.6	1.00	288
MiBP	2005–2006	5.07	8.81	15.2	21.3	5.00	2.4	1.00	7.3
MnBP	2005–2006	18.3	30.8	50.8	77.8	17.7	2.4	0.99	26
MP	2005–2006	58.8	221.0	527.0	902.0	63.7	5.2	0.99	247
Triclosan	2003–2004	9.48	43.9	212.0	368.0	9.67	9.8	1.00	131
Abbreviations: MBzP, monobenzyl phthalate; MECPP, mono(2-ethyl-5-carboxypentyl) phthalate; MEHHP, mono(2-ethyl-5-hydroxyhexyl) phthalate; MEHP, mono(2-ethylhexyl) phthalate; MEOHP, mono(2-ethyl-5-oxohexyl) phthalate; MiBP, mono(isobutyl) phthalate; MnBP; mono(n-butyl) phthalate. aThe z-scores (standard scores) used in the regression were 0.00 for the 50th, 0.67 for the 75th, 1.28 for the 90th, and 1.64 for the 95th percentiles. bUrinary levels of BPA include both conjugated and unconjugated forms.																		

We augmented the primary assessment of population intake rates for IPR calculations by repeating the analysis for all years for which analyte excretion data are available. A goal of that exercise was to explore the extent to which the per capita average excretion rates have been varying with time. The earliest data are from the 1999–2000 period and for some analytes, monitoring data are available on a biennial basis for five consecutive periods through 2008. For other analytes, biomonitoring began more recently as in the case of MP, for which the first data are for 2005–2006. For the full analysis of per capita average intake rates based on urinary excretion data, see Supplemental Material, Table S3 (http://dx.doi.org/10.1289/ehp.1204992).

We estimated the population average intake rates of parent compounds from the AM analyte levels in urine, taking into account empirical data on fractional excretion of the analytes following ingestion exposure and also accounting for the relative molecular weights of the analytes and the parent compounds. For three of the phthalates (BBzP, DEHP, and DnBP) and their associated monoester metabolites, we relied on the summary data for molar excretion rates as reviewed by Wittassek et al. (2011). For the other two phthalates (DiBP and DEP), lacking direct experimental data, we assumed that the fractional excretion of the associated metabolites was the same as for DnBP. For BPA, we assumed complete urinary excretion, on the basis of the report by Dekant and Völkel (2008). For DCB, we also assumed complete excretion of the associated metabolite DCP on the basis of an exposure study of Yoshida et al. (2002). For triclosan, we assumed 57% urinary excretion, on the basis of the work of Sandborgh-Englund et al. (2006). We were unable to locate any published information on the fractional urinary excretion of MP, and so we conducted the analysis assuming that the upper bound must be complete excretion. Table 2 summarizes the fractional urinary excretion estimates as well as the molecular weights used in the analysis.

**Table 2:** Fractional molar urinary excretion factors (f_ue_) and relevant molecular weights (MW) for converting excretion rates to intake rates.

Parent compound	MW (g/mol)	Analyte	MW (g/mol)	Urinary excretion, *f*_ue_	Reference for f_ue_
BPA	228	BPA	228	1.0	Dekant and Völkel 2008
BBzP	312	MBzP	256	0.73	Anderson et al. 2001
DEHP	391	MEHP	294	0.059	Koch et al. 2005
MEHHP	294	0.233	Koch et al. 2005
MEOHP	292	0.15	Koch et al. 2005
MECPP	308	0.185	Koch et al. 2005
DEP	222	MEP	194	0.69	(Assumed same as DnBP)
DiBP	278	MiBP	222	0.69	(Assumed same as DnBP)
DnBP	278	MnBP	222	0.69	Anderson et al. 2001
DCB	147	DCP	163	1.0	Yoshida et al. 2002a
MP	152	MP	152	< 1.0	(Upper bound)
Triclosan	290	Triclosan	290	0.57	Sandborgh-Englund et al. 2006
Abbreviations: MBzP, monobenzyl phthalate; MECPP, mono(2-ethyl-5-carboxypentyl) phthalate; MEHHP, mono(2-ethyl-5-hydroxyhexyl) phthalate; MEHP, mono(2-ethylhexyl) phthalate; MEOHP, mono(2-ethyl-5-oxohexyl) phthalate; MiBP, mono(isobutyl) phthalate; MnBP; mono(n-butyl) phthalate. aPersonal monitoring and urinary excretion data, for ordinary environmental inhalation exposures among 119 adults in Osaka, Japan, are consistent with fue = 1.0.										

Two other data elements were required to complete the analysis: creatinine clearance rates and body masses. For the former, we used 18 mg/kg/day for females and 23 mg/kg/day for males, following similar calculations by Fromme et al. (2007) and Kohn et al. (2000). For body masses, we computed U.S. population average values by applying the age-specific data for 2002 reported in Ogden et al. (2004). The mean was estimated separately for males and for females as the evenly weighted mean for all ages between 6 and 69 years of age. The results were 69.2 kg for females and 79.5 kg for males. Combining these factors, we estimated an average urinary creatinine clearance rate of 1.25 g/day for females and 1.83 g/day for males, or 1.54 g/day as the combined population average.

For the case of a single analyte resulting from a given parent compound, the population-mean daily intake of the parent compound is estimated from Equation 2 (Kohn et al. 2000):


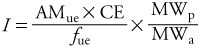
[2]

where *I* is the daily average intake (micrograms per day per person), AM_ue_ is the AM level excreted in urine (micrograms per gram creatnine; Table 1), CE is the population mean creatinine excretion rate (1.54 g/day per person), *f*_ue_ is the urinary excretion fraction (Table 2), and MW_p_ and MW_a_ are the respective molecular weights of the parent compound and of the urinary analyte (Table 2).

For DEHP, with four analytes, a modified approach was used to obtain an overall estimate:


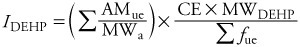
[3]

In applying Equation 3, the sums were separately evaluated for the four primary metabolites and the results substituted into the equation to obtain an estimate of DEHP intake.

In using urinary metabolites as quantitative markers of intake, it is important to recognize the possibility that certain metabolites may have multiple potential parent compounds. We have relied on information from the biomonitoring report (CDC 2012), which indicates that the metabolites considered in this study have a single parent compound with one exception. Mono(*n*-butyl) phthalate (MnBP) is a metabolite of both DnBP and BBzP. In the analysis presented here, we have assigned the MnBP data entirely to DnBP intake on the basis of two lines of evidence. First, Anderson et al. (2001) have shown that BBzP is converted to monobenzyl phthalate (MBzP) with a conversion rate that is > 10× as large as for MnBP. Second, the average urinary level of MnBP is roughly twice the level of MBzP. Consequently, the error in assuming that all MnBP is attributable to DnBP intake is no larger than 5%, which is negligible in the current context.

Variability in the biomonitoring data provides indicators of uncertainty in the intake estimates. For most chemicals, relative to the uncertainty in published production data, intake uncertainty is small. For this study, we estimated the percentage uncertainty in daily per capita intake as the larger of *a*) the square root of the ratio of maximum to minimum in the reported range of GM excretion rates as reported by the CDC (2012), and *b*) the relative SD in year-to-year AM excretion rates. These values were then combined in quadrature with the uncertainty estimates for production data to obtain an overall estimated uncertainty in the IPR [see Supplemental Material, Equation S1 (http://dx.doi.org/10.1289/ehp.1204992)]. For triclosan and MP, only lower and upper bounds are determined for the IPR because the data are not well suited for evaluating best point estimates. (For details of the uncertainty analysis, see Supplemental Material, pp. 4–6 and Table S4.)

## Results and Discussion

**Figure 1:**
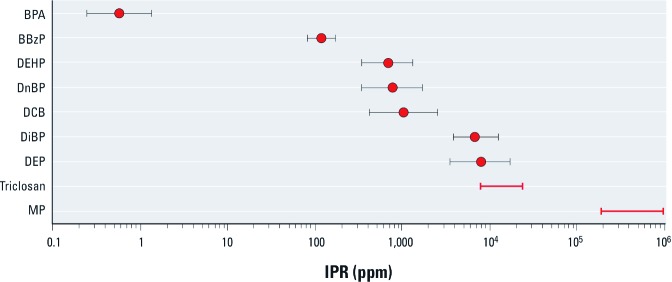
IPR estimates for the U.S. population for nine manufactured chemicals. A value of 1 ppm indicates that the aggregate intake, summed across the population, is 1 g for every million grams manufactured in or imported into the population’s economy. Error bars indicate estimated uncertainty for BPA, BBzP, DEHP, DnBP, DCb, DiBP, and DEP; red bars indicate lower and upper bounds of the estimated ranges for triclosan and MP.

We estimated IPR values for recent conditions in the United States for nine common manufactured chemicals (Table 3, Figure 1). A striking feature of the results is the very broad range of IPR values obtained, spanning more than five orders of magnitude from 0.6 ppm for BPA to > 180,000 ppm for MP. The probable explanation for this broad range is that different uses of these chemicals lead to very different degrees of opportunity for exposure, or different levels of exposure intimacy, as expressed through intake into the human body. For example, a main use of BPA is as a starting material in the manufacture of polycarbonate plastics and epoxy resins, accounting for 72% and 21%, respectively, of the BPA consumed in the United States in 2003 (NTP 2007). In these products, most of the original BPA is transformed into the polymeric fabric of the material. Small amounts of monomer may be released after manufacture either owing to the presence of residual monomer or as the polycarbonate or epoxy degrades (Kang et al. 2006). Consequently, one expects that human exposure to BPA would be limited to a small fraction of the original BPA used to produce the polycarbonate plastic or the epoxy resins. The end products made from BPA also have widely variable human exposure potential, from high potential in food-can linings to low potential in auto-body parts.

**Table 3:** IPR for selected chemicals in the United States.

Chemical	CAS No.	Intake (µg/day per person)a	Production (g/day per person)b	IPR (ppm)
BPA	80-05-7	5.6	9.8	0.6
BBzP	85-68-7	35	0.30	120
DEHP	117-81-7	550	0.82	670
DnBP	84-74-2	71	0.094	760
DCB	106-46-7	97	0.094	1,040
DiBP	84-69-5	20	0.0030	6,800
DEP	84-66-2	730	0.094	7,700
Triclosan	3380-34-5	350	0.014–0.044	8,000–24,000
MP	99-76-3	> 380	< 0.0021	> 180,000
aPer capita average daily intake of the chemical across the U.S. population. bPer capita average daily production plus importation rate of chemical for the U.S. economy.								

Conversely, the relatively high IPR for triclosan, an antibacterial agent, very likely reflects its major use in personal care products. Many brands of toothpaste, antibacterial soaps, and mouthwashes use triclosan as an active ingredient. Exposures as a consequence of ingestion (toothpaste or mouthwash) or dermal permeation (soaps) would be relatively direct. The high IPR for triclosan suggests that these uses lead to intakes that are a meaningful fraction of the total amount of triclosan incorporated into personal care products.

Similarly, MP is “widely used as a preservative in food, cosmetic and pharmaceutical products” (Soni et al. 2005). Its use in food and in ingested pharmaceuticals would be associated with an expected IPR approaching unity (i.e., 1,000,000 ppm). As a component of cosmetics, there is opportunity for intake via at least three pathways: transdermal permeation, inhalation of the semivolatile compound, and incidental ingestion. MP has some natural sources; however, Soni et al. (2005) reported that, “paraben intake from natural sources is negligible.”

As illustrated by these cases, the IPR can vary over several orders of magnitude, with the degree of variability depending on the ways in which the chemical is used. As noted above, a chemical primarily used in products that are ingested should have an IPR approaching 1,000,000 ppm. To elaborate, if all of a manufactured chemical is added to food that is ingested, then the cumulative intake in a human population would be the same as the cumulative production of the chemical. The IPR for a chemical mainly used externally in personal care products might be somewhat smaller, perhaps on the order of 10,000–100,000 ppm. The IPR for a chemical that is chiefly used as a constituent of sprays or pesticides applied indoors might be in the range of 100–10,000 ppm. The IPR for SVOCs present as additives in polymeric products, such as plasticizers or flame retardants, might be on the order of 1–1,000 ppm. We caution that these are rough estimates that are guided in part by the limited IPR investigations reported here as well as prior studies of iFs for indoor pollutant emissions (Nazaroff 2008) and research on the dynamic behavior of indoor SVOCs, as reviewed by Weschler and Nazaroff (2008). Further studies that better characterize IPRs, not only in these but also in other use categories, appear to be warranted.

Among possible applications, we envision that the IPR might be a useful metric for preliminary screening for some chemicals in the following manner. There are on the order of 30,000 commercial chemicals to prioritize in terms of public exposure. Where data permit, the reported or intended use of a compound could inform its assignment to a specific IPR range or bin. Combining available information on annual production with the chemical’s assigned IPR range would yield a preliminary estimate of the anticipated range for the population’s intake rate for that chemical. Such zero-order screening would allow those compounds with the highest anticipated intake rates to be identified, which could be of value in prioritizing chemicals for more detailed health risk assessments.

As detailed in Supplemental Material, Table S4 (http://dx.doi.org/10.1289/ehp.1204992), the uncertainty in the estimates reported here is dominated by the lack of precision in the production data. For five of the compounds studied here (BPA, DEHP, DnBP, DCB, and DEP), production uncertainty contributes a multiplicative uncertainty of 2.2–2.3×. For BBzP and DiBP, the production uncertainty is smaller owing to a narrower reporting range, and particularly in the case of DiBP, the intake uncertainty makes a meaningful contribution to the overall uncertainty estimate. For triclosan and for MP, we were only able to estimate ranges that bracket the expected IPR values. For triclosan, the range spans a factor of three, accounting for the uncertainty in production in 2002 based on data reported for the period 1986–1998. For MP, the production rate is expressed as an upper bound. The intake estimate represents a lower bound based on the assumption that urinary excretion is complete. The consequence is an IPR estimate (180,000 ppm) that represents a lower bound. The upper bound cannot exceed 1,000,000 ppm, so the IPR for MP can be bracketed within a range that spans a factor of five. Importantly, the uncertainties, while moderately high, are small compared to the differences in IPR estimates for these chemicals (Figure 1). Consequently, we believe that there is useful information in the IPR estimates despite the moderately high uncertainties.

Because IPR is defined as the ratio of two rates, temporal aspects merit consideration. The IPR is best suited for characterizing exposure to chemicals that move fairly rapidly from manufacture to human intake. The IPR would also be well suited for cases in which the production rates and intake rates are relatively stable over time. Consider, for example, DEHP as a plasticizer. To the extent that it is used in products such as vinyl flooring, DEHP would be expected to be persistent, contributing to exposure for years or decades after manufacture. Intake in any given year would result not only from the DEHP manufactured in that year but also from DEHP manufactured in prior years that is present in products that are still in use. Interestingly, though, a study of German university students showed strong year-by-year tracking between economy-wide production data and estimated intake rates for the period 1988–2004 (Helm 2007). Wittassek et al. (2011) have reported an analogous finding for DnBP. We made estimates of the IPR values for these two chemicals in Germany, based on data extracted from Figure 3 of Wittassek et al. (2011). The results indicate IPR values in the ranges of 30–60 ppm for DEHP and 500–800 ppm for DnBP. The DEHP result is about an order of magnitude lower than our estimate for the U.S. population, potentially reflecting earlier restrictions on the use of DEHP in Europe than in the United States (Europa 1999). The DnBP results are about the same for the U.S. and German populations.

There are sufficient biomonitoring and chemical production data in the United States to separately estimate IPR values for 2001–2002 and for 2005–2006 for the five phthalates considered. For the report of the results of that assessment, see Supplemental Material, Table S5 (http://dx.doi.org/10.1289/ehp.1204992). Overall, the results show reasonable consistency over time: the average deviation across the five species between the two time periods is 34%.

A general challenge in using biomonitoring data for IPR assessments is to relate measured levels in biological fluids to rates of intake. The IPR concept is not restricted to chemicals excreted in urine after human intake. To the extent that levels in blood, breath, or other media can be used to infer intake, these metrics, sampled in representative subpopulations, could be used to estimate IPRs. For the chemicals considered here, the rapid urinary excretion of the primary chemicals and/or their metabolites enables a relatively simple quantitative description of this relationship. For bioaccumulative compounds, levels measured in body fluids can be translated into intake estimates through pharmacokinetic models (e.g., Hays and Aylward 2012; Lorber 2008; Lorber and Egeghy 2011).

It is also possible to estimate IPR values from historic data. Consider the example of pentachlorophenol, a wood preservative and biocide widely used in the United States prior to regulatory changes in the mid-1980s. Data presented by Hattemer-Frey and Travis (1989) support an IPR estimate for pentachlorophenol in the U.S. population during the late 1980s of 60 ppm, based on an annual production of 23 million kg, an estimated average intake of 16 µg/day per person, and a U.S. population at the time of 240 million. In the context of the results presented in Figure 1, this estimate suggests a low-to-intermediate level of exposure intimacy for the historic uses of pentachlorophenol, higher than current values for BPA and lower than estimated values for the phthalates.

The IPR complements another aggregate exposure metric, the iF (Bennett et al. 2002b). Both are dimensionless ratios quantifying exposure intensity. The numerator in each case represents an intake summed over an exposed population. However, the denominators are distinct, with the denominator of the iF quantifying an environmental release, whereas the IPR denominator measures the total quantity of a chemical manufactured and/or imported. The IPR is necessarily applicable only over large (economy-wide) populations, whereas the iF can be applied more flexibly across widely variable population sizes. The iF is well suited for characterizing exposure to chemicals that are created incidentally, such as combustion by-products. The IPR is well suited for characterizing exposure to chemicals that are synthesized and used either neat (e.g., certain pesticides) or as product constituents, especially those that partition significantly among multiple phases.

## Conclusions

The IPR is an aggregate metric of exposure intensity that is appropriate for characterizing population-level exposure to synthesized chemicals, particularly those that move fairly rapidly from manufacture to human intake and for which production and intake rates are relatively stable.

Novel insights concerning public exposures to manufactured chemicals can be gained from further development and application of the IPR metric. For example, collections of IPRs for manufactured chemicals with a range of properties and uses could inform assessment of potential exposures for new or data-poor chemicals. Statistical modeling approaches could be used to capture aggregate information embedded in the IPRs and these results could be combined with predictions from mechanistic models to improve estimates of potential exposure. When combined with rapid toxicity screening tools (Judson et al. 2010, 2011; Rotroff et al. 2010), methods to rapidly estimate potential for human exposure could contribute to improved health-risk-based prioritization of a wide range of chemicals of concern (Cohen Hubal et al. 2010; Egeghy et al. 2012). The chemical/source combinations of greatest concern can then be more comprehensively investigated. Because our understanding of the vast number of sources and wide range of exposure pathways remains rudimentary for many manufactured chemicals, it is clear that an iterative approach will be required. As the mechanisms and pathways controlling the source-to-dose continuum are more clearly elucidated, improved methods to obtain rapid screening-level exposure estimates should emerge.

## Supplemental Material

(217 KB) PDF
