# Supplementary material for: Intake to Production Ratio: A Measure of Exposure Intimacy for Manufactured Chemicals
Source: Environ Health Perspect. 2012 Sep 25;120(12):1678–83. doi: 10.1289/ehp.1204992 (PMC3546365; doi:10.1289/ehp.1204992)
Supplement: (217 KB) PDF [file ehp.1204992.s001.pdf]

Supplemental Material

Intake to Production Ratio: A Measure of Exposure Intimacy for Manufactured Chemicals

William W. Nazaroff[1], Charles J. Weschler[2,3], John C. Little[4], Elaine A. Cohen Hubal[5]

[1] Department of Civil and Environmental Engineering, University of California, Berkeley, California, USA
[2] Environmental and Occupational Health Sciences Institute, University of Medicine & Dentistry of New Jersey & Rutgers University, Piscataway, New Jersey, USA
[3] International Centre for Indoor Environment & Energy, Technical University of Denmark, Lyngby, DK
[4] Department of Civil and Environmental Engineering, Virginia Tech, Blacksburg, Virginia, USA
[5] National Center for Computational Toxicology, Environmental Protection Agency, Research Triangle Park, North Carolina, USA

Table of Contents for Supplemental Materials

Time-dependent per-capita production rates of selected manufactured chemicals 1

Time-dependent urinary excretion data 3

Uncertainty estimates for production, intake, and IPR.. 4

Comparing IPR estimates for five phthalates: 2001-2002 versus 2005-2006 6

References 7

Time-dependent per-capita production rates of selected manufactured chemicals

The USEPA Inventory Update Reporting (IUR) and Chemical Data Reporting system makes available chemical production data at four-year reporting intervals beginning in 1986. The most recent publicly available data are from the 2006 reporting year. Table S1 presents data extracted from this system for the nine chemicals investigated in the present research. Wherever possible, these data are used to provide point estimates of per-capita daily production rates, as summarized in Table S2. In a few instances, the per capita emission rates are estimated based either on data that were obtained outside the IUR system or based on extrapolation. In particular,

the entries for year 2006 for BPA and for DEHP are based on more precise reporting of production data as detailed in the footnotes. For triclosan in 2002, there are no data reported in the IUR. We have estimated the production rate in that year as bounded between two values. The lower bound (0.014 g/d per person) assumes no change in the per capita production rate from 1998. The upper bound (0.044 g/d per person) is based on an exponential growth model fit to and extrapolated from the reported data for 1986 through 1998.

Table S1. US chemical production data (pounds per year) by reporting year (USEPA 2012) [a]

**Species**	**CAS No.**	**1986**	**1990**	**1994**	**1998**	**2002**	**2006**
BPA	80-05-7	0.5-1 B	> 1 B	> 1 B	> 1 B	> 1 B	> 1 B
BBzP	85-68-7	50-100 M	100-500 M	50-100 M	100-500 M	50-100 M	50-100 M
DEHP	117-81-7	100-500 M	100-500 M	100-500 M	100-500 M	100-500 M	100-500 M
DnBP	84-74-2	10-50 M	10-50 M	10-50 M	10-50 M	10-50 M	10-50 M
DCB	106-46-7	10-50 M	50-100 M	50-100 M	50-100 M	50-100 M	10-50 M
DiBP	84-69-5	1-10 M	1-10 M	1-10 M	1-10 M	0.5-1 M	0.5-1 M
DEP	84-66-2	10-50 M	10-50 M	10-50 M	10-50 M	10-50 M	10-50 M
Triclosan	3380-34-5	0.01-0.5 M	0.01-0.5 M	0.5-1 M	1-10 M	NR	NR
MP	99-76-3	0.5-1 M	1-10 M	1-10 M	0.5-1 M	0.01-0.5 M	< 0.5 M

[a] Abbreviations: BPA – bisphenol A; BBzP – butyl benzyl phthalate; DEHP – di(2-ethylhexyl) phthalate; DnBP – di(n-butyl) phthalate; DCB – para-dichlorobenzene; DiBP – di(isobutyl) phthalate; DEP – diethyl phthalate; MP – methyl paraben; M – million (10^6); B – billion (10^9); NR – not reported.

Table S2. Estimated per-capita chemical production rate (g/d per person) for the United States.[a]

**Species**	**1986**	**1990**	**1994**	**1998**	**2002**	**2006**
BPA	3.7	> 5.0	> 4.8	> 4.6	> 4.4	9.8 [b]
BBzP	0.37	1.1	0.34	1.0	0.31	0.30
DEHP	1.2	1.1	1.1	1.0	0.98	0.82 [c]
DnBP	0.12	0.11	0.11	0.10	0.10	0.094
DCB	0.12	0.36	0.34	0.32	0.31	0.094
DiBP	0.017	0.016	0.015	0.014	0.003	0.0030
DEP	0.12	0.11	0.11	0.10	0.10	0.094
Triclosan	0.0004	0.0004	0.0034	0.014	0.014-0.044 [d]	n/a
MP	0.004	0.016	0.015	0.003	0.0003	< 0.0021

[a] Abbreviations: BPA – bisphenol A; BBzP – butyl benzyl phthalate; DEHP – di(2-ethylhexyl) phthalate; DnBP – di(n-butyl) phthalate; DCB – para-dichlorobenzene; DiBP – di(isobutyl) phthalate; DEP – diethyl phthalate; MP – methyl paraben; n/a – not available. US population by year (millions): 238 (1986), 247 (1990), 260 (1994), 273 (1998), 285 (2002), 295.5 (2006).
[b] Applicable to the year 2004, based on a reporting of 2.3 billion lbs/y of production (NTP 2007).
[c] Based on a reporting of 194 million lbs/y of production (NTP 2011).
[d] Lower bound assumes no growth from 1998; upper bound is based on a fit of exponential growth at a constant rate for the data from 1986, 1990, 1994, and 1998.

Time-dependent urinary excretion data

Table S3 summarizes all of the available data on urinary analyte concentrations for the nine chemicals assessed in this paper. The four percentile results for each species are as reported by the CDC (2012) for each indicated two-year sampling interval. The geometric mean (GM) and geometric standard deviation (GSD) are determined from regression analysis applied to the percentile results, assuming that the distributions are lognormal. The high coefficient of determination (r^2 consistently close to 1.0) indicates that the lognormal model for the distribution is reasonable across the C_{50} to C_{95} range. The arithmetic mean (AM) analyte concentration is computed from the GM and GSD according to equation [1]. A clear trend with time is exhibited only for MiBP, which increases in each successive study period and changes by 2.6× between 2001-2002 and 2007-2008. There may be a decrease in MBzP from 2001-2002 to 2007-2008, but the trend is not as clear as for MiBP. All of the other species exhibit moderate variability in AM values among study periods, but display no clear trend. The median relative standard deviation for the temporal variability in AM values is 15% (range: 3% to 40%).

Table S3. Computed arithmetic mean analyte levels in urine (µg/g creatinine) for US population (aged ≥ 6). [a]

**Analyte**	**Year**	C_{50}	C_{75}	C_{90}	C_{95}	**GM**	**GSD**	r^2	**AM**
MEHP	99-00	3.08	5.88	10.8	18.9	2.95	2.94	0.99	5.3
MEHP	01-02	3.9	7.94	18	32.8	3.65	3.62	0.99	8.4
MEHP	03-04	1.89	4.31	10.8	25.4	1.71	4.69	0.98	5.6
MEHP	05-06	2.61	5.69	13.7	30.1	2.38	4.30	0.98	6.9
MEHP	07-08	2.36	5.15	11.8	21.9	2.23	3.83	0.99	5.5
MEHHP	01-02	16.6	32.2	71.1	143	15.16	3.62	0.98	34.7
MEHHP	03-04	17.7	35.8	93.5	182	16.01	4.12	0.99	43.7
MEHHP	05-06	21.4	46.1	117	235	19.62	4.25	0.99	55.9
MEHHP	07-08	19.3	40.5	99.3	179	18.02	3.88	0.99	45.2
MEOHP	01-02	11.2	21.3	45.2	87	10.33	3.41	0.98	21.9
MEOHP	03-04	12.1	24.3	63	118	11.02	4.01	0.99	28.9
MEOHP	05-06	13.5	28.9	77.7	144	12.43	4.25	0.99	35.4
MEOHP	07-08	11	22.3	52.9	107	10.03	3.92	0.98	25.5
MECPP	03-04	27	54.6	139	251	24.85	3.90	0.99	62.8

MECPP	05-06	32.2	67.5	168	290	30.23	3.84	0.99	74.7
MECPP	07-08	29.1	58.7	138	233	27.42	3.56	0.99	61.5
MiBP	01-02	2.46	4.54	8.02	12	2.42	2.60	1.00	3.8
MiBP	03-04	3.57	6.21	10.9	15.4	3.51	2.43	1.00	5.2
MiBP	05-06	5.07	8.81	15.2	21.3	5.00	2.39	1.00	7.3
MiBP	07-08	7.25	12.1	19.5	27.8	7.13	2.24	1.00	9.9
MnBP	01-02	17.4	30.3	52.4	81.3	16.84	2.52	0.99	25.8
MnBP	03-04	19.3	33.9	59	91.6	18.70	2.55	0.99	28.9
MnBP	05-06	18.3	30.8	50.8	77.8	17.74	2.37	0.99	25.8
MnBP	07-08	18.8	31.4	52	77.1	18.29	2.33	1.00	26.2
MBzP	99-00	9.6	18.1	36.1	55.7	9.28	2.92	1.00	16.4
MBzP	01-02	9.71	19.1	39.5	65.1	9.31	3.17	1.00	18.1
MBzP	03-04	9.04	17.7	33.1	50.4	8.92	2.83	1.00	15.3
MBzP	05-06	8.24	15.3	30.2	47.4	7.92	2.89	1.00	13.9
MBzP	07-08	7.2	14.3	28.3	42.6	7.08	2.95	1.00	12.7
BPA	03-04	2.5	4.29	7.67	11.2	2.43	2.49	1.00	3.7
BPA	05-06	1.71	3.01	5.73	9.7	1.61	2.83	0.99	2.8
BPA	07-08	1.95	3.45	6.09	10	1.87	2.65	0.99	3.0
Triclosan	03-04	9.48	43.9	212	368	9.67	9.80	1.00	131
Triclosan	05-06	13	73.2	304	532	14.17	9.88	0.99	195
Triclosan	07-08	12.4	50.4	233	443	12.16	9.20	1.00	143
MEP	99-00	93	237	598	1290	87.58	4.82	0.99	302
MEP	01-02	97.4	256	640	1220	94.59	4.60	1.00	303
MEP	03-04	101	298	733	1350	101.61	4.78	1.00	345
MEP	05-06	92.3	242	625	1140	89.86	4.60	1.00	288
MEP	07-08	75.3	204	547	987	73.59	4.79	1.00	251
DCP	03-04	9.29	34.4	141	578	7.91	11.53	0.98	157
DCP	05-06	7.32	20.4	89.3	292	6.02	9.22	0.98	70.8
DCP	07-08	6.24	24.2	109	409	5.38	12.15	0.99	122
MP	05-06	58.8	221	527	902	63.68	5.19	0.99	247
MP	07-08	61	228	538	820	67.47	4.86	0.99	235

[a] The *z*-scores (standard scores) used in the regression were 0.00 for C_{50}, 0.67 for C_{75}, 1.28 for C_{90}, and 1.64 for C_{95}. The analyte abbreviations refer to the following chemicals — bisphenol A (BPA), 2,5-dichlorophenol (DCP), mono-benzyl phthalate (MBzP), mono-2-ethylhexyl phthalate (MEHP), mono-(2-ethyl-5-hydroxyhexyl) phthalate (MEHHP), mono-(2-ethyl-5-oxohexyl) phthalate (MEOHP), mono-(2-ethyl-5-carboxypentyl) phthalate (MECPP), mono-ethyl phthalate (MEP), mono-isobutyl phthalate (MiBP), mono-n-butyl phthalate (MnBP), and methyl paraben (MP). Urinary levels of bisphenol A include both conjugated and unconjugated forms.

Uncertainty estimates for production, intake, and IPR

Available data permit uncertainties to be estimated but not rigorously evaluated. We apply a standard propagation of errors approach, beginning with the defining equation, IPR = I/P

(where I = per capita intake rate and P = per capita production rate), assuming that I and P are independent, and assuming that the parameters are lognormally distributed. The uncertainties in log(I) and log(P) are added in quadrature to obtain the uncertainty in log(IPR):

$$\sigma_{\log(\mathrm{IPR})} = \left[\left(\sigma_{\log(I)} \right)^2 + \left(\sigma_{\log(P)} \right)^2 \right]^{1/2} \qquad \text{[S.1]}$$

For most species, the uncertainty in log(P) is estimated as half of the production rate range as indicated in Table S1. For example, for DEP in 2006, the difference between log(50 M) and log (10 M) is 0.70, so the estimated uncertainty in log(P) is 0.35, indicating that the uncertainty in P is a multiplicative factor of 2.2 (= $10^{0.35}$). For bisphenol A, the uncertainty is estimated as the ratio of the point estimate (2.3 billion) and the IUR lower bound (1 billion), a factor of 2.3. Similarly, for DEHP, the uncertainty is estimated as the ratio of the point estimate (194 M) and the nearest bound in the IUR range (100 M), a factor of 1.9. For triclosan, production is assumed to lie between the upper and lower bounds as described in the first section of this Supplemental Material. For methyl paraben, the production value reported in the IUR is treated as an upper bound.

For intake, the uncertainty is estimated using the time-series data reported in Table S3. For seven of the nine chemicals, the multiplicative uncertainty is estimated as the square root of the maximum to minimum arithmetic mean urinary excretion rate of the target analyte. For DEHP, the same basic approach is applied, but the four target metabolites are first combined using the fractional urinary excretion (Table 2) as a weighting factor. For methyl paraben, lacking good information on the fractional clearance by urine, we assume complete excretion and treat the resulting intake estimate as an upper bound.

Table S4 summarizes the results, showing that only in the case of DiBP does intake uncertainty make a strong contribution to uncertainty in the IPR relative to the uncertainty in

production rates. The uncertainty indicators are plotted as error bars in Figure 1 of the main paper. Because of the necessarily ad-hoc procedure for estimating uncertainty, the meaning of these bars is not precisely known. Given the manner in which they have been constructed, a reasonable approximation might be to consider them as ~ 90% confidence limits.

Table S4. Estimated uncertainties for intake, production, and the intake-to-production ratio.

**Chemical**	**Intake**	**Production**	**IPR**
Bisphenol A (BPA)	1.2×	2.3×	2.3×
butyl benzyl phthalate (BBzP)	1.2×	1.4×	1.5×
di(2-ethylhexyl) phthalate (DEHP)	1.1×	1.9×	2.0×
di(n-butyl) phthalate (DnBP)	1.1×	2.2×	2.2×
para-dichlorobenzene (DCB)	1.5×	2.2×	2.4×
di(isobutyl) phthalate (DiBP)	1.6×	1.4×	1.8×
diethyl phthalate (DEP)	1.2×	2.2×	2.2×
Triclosan	1.2×	Bracketed range	Bracketed range
methyl paraben	Lower bound	Upper bound	Lower bound

Comparing IPR Estimates for Five Phthalates: 2001-2002 versus 2005-2006

For the five phthalates considered in this study, sufficient information is available to estimate IPR values for two separate time periods, 2001-2002 and 2005-2006. The results are presented in Table S5. For three of the species (BBzP, DnBP, and DEP), the results are highly consistent between the two time periods. For the other two chemicals (DEHP and DiBP), the estimated IPRs for the 2005-2006 period are about 2× higher than for the 2001-2002 period. These differences may reflect uncertainty in the IPR estimates (they are comparable to the uncertainty scale estimated in Table S4). There also may be true changes in IPR values over time if the proportions of use shift among applications with strongly different exposure intimacies. Available information is insufficient to distinguish the causes in these two cases.

Table S5. Intake-to-production ratio (IPR) for phthalates using 2001-2002 data (01-02) compared with the IPR derived from 2005-2006 data (05-06).

**Chemical**	***I*** **(µg/d per person)**	***P*** **(g/d per person)**	**01-02 IPR (ppm)**	**05-06 IPR (ppm)** [a]
butyl benzyl phthalate (BBzP)	46	0.31	150	120
di(2-ethylhexyl) phthalate (DEHP)	300 [b]	0.98	310	670
di(n-butyl) phthalate (DnBP)	71	0.10	730	760
di(isobutyl) phthalate (DiBP)	11	0.003	3400	6800
diethyl phthalate (DEP)	770	0.10	7900	7700

[a] Reproduced from Table 3 of the paper.
[b] Analysis based on three metabolites (MEHP, MEHHP, and MEOHP) as monitoring of MECPP did not begin until the 2003-2004 survey period.

References

CDC [Centers for Disease Control and Prevention]. 2012. National Report on Human Exposure to Environmental Chemicals. Available: http://www.cdc.gov/exposurereport [accessed 24 May 2012].

NTP (National Toxicology Program). 2007. NTP-CERHR Expert Panel Report on the Reproductive and Developmental Toxicity of Bisphenol A. Report NTP-CERHR-BPA-07. Available: cerhr.niehs.nih.gov/evals/bisphenol/BPAFinalEPVF112607.pdf [accessed 19 January 2012].

NTP (National Toxicology Program). 2011. Report on Carcinogens. Twelfth Edition. Available: http://ntp.niehs.nih.gov/?objectid=03C9AF75-E1BF-FF40-DBA9EC0928DF8B15 [accessed 24 May 2012].

US Census. 2001. Resident Population Estimates of the United States by Sex, Race, and Hispanic Origin: April 1, 1990 to July 1, 1999, with Short-Term Projection to November 1, 2000. Available: http://www.census.gov/population/estimates/nation/intfile3-1.txt [accessed 24 May 2012].

US Census. 2011. Intercensal Estimates of the Resident Population by Sex and Age for the United States: April 1, 2000 to July 1, 2010 (US-ESTINT-01) Available: http://www.census.gov/popest/data/intercensal/national/nat2010.html [accessed 24 May 2012].
